# Analysis on spatial structure characteristics and influencing factors of Beijing-Tianjin-Hebei Region MICE cities

**DOI:** 10.1371/journal.pone.0287695

**Published:** 2023-07-18

**Authors:** Shifeng Wu, Yu Hong, Yinuo Jia, Jiangyun Wang, Xin Xu

**Affiliations:** 1 Research Center for Green Development of Great Wall Cultural Economic Belt, Hebei University of Economics and Business, Shijiazhuang, China; 2 School of Tourism, Hebei University of Economics and Business, Shijiazhuang, China; 3 School of Tourism, Hainan University, Haikou, China; Shenzhen University, CHINA

## Abstract

Industrial upgrading and transfer is one of the three key areas in the coordinated development of Beijing-Tianjin-Hebei Region. Meetings, incentives, conferences and exhibitions (MICE) are important means of promoting industrial upgrading. Urban MICE industry, as an important carrier of event activities, become an important gripper for coordinated development of Beijing-Tianjin-Hebei Region. City exhibition space structure plays an dominant role in regional economic development, and it will greatly promote smooth implementation of the coordinated development strategy of Beijing-Tianjin-Hebei Region. In this paper, 13 prefecture-level cities in Beijing, Tianjin and Hebei were selected as research objects, and the data from 2012 to 2018 were selected to establish a gravity model of the attractiveness of MICE cities. With the help of UCINET software, the network density, centrality, cohesive subgroup and core-edge of nodes researches were obtained to analyze the spatial structure characteristics of the attractiveness of MICE cities in Beijing-Tianjin-Hebei Region. The influencing factors of the spatial characteristics of MICE city attractiveness are analyzed by using geographically weighted regression model. The results show that: (1) Beijing, as the overall core area, act as a prominent role. Beijing strengthens the attraction to the superior resources of the surrounding areas, but the network of MICE cities tends to be unbalanced. (2) Overall space forms a subgroup from a single independent subgroup to a subgroup that is spatially separated from each other and acts as an intermediary channel to connect each other, and the core region decreases from 3 to 2. Langfang was removed from the list, leaving Beijing and Tianjin as the core. (3) The influence of supporting facilities, urban environment and population factors on the MICE city attractiveness of Beijing-Tianjin-Hebei Region is increasing gradually. The influence of tourism development level on the MICE city attractiveness of Beijing-Tianjin-Hebei Region is decreasing gradually. The influence of economic development level and Internet development level on the MICE city attractiveness of Beijing-Tianjin-Hebei Region remains unchanged.

## Introduction

Coordinated Development of the Beijing-Tianjin-Hebei Region (BTH Coordinated Development) is a major strategic deployment following the development trend of the new era, and focusing on the overall situation of China’s development [1–2]. Industrial upgrading and transfer is one of the three key areas of BTH Coordinated Development. Current researches about BTH Coordinated Development mainly focus on policy formulation and evaluation, and key areas like transportation logistics, manufacturing industry, tourism economy and their industrial spatial structure [3–10]. MICE industry is an important means to promote urban industrial upgrading. As an important carrier of events activities, urban MICE industry has become an important way for the BTH Coordinated Development. The spatial structure of urban MICE activities play an important role in regional economic development and promoting smooth implementation of BTH Coordinated Development [11–17]. During the ‘13th Five Year Plan’ period, the competition of urban industrial clusters is increasingly fierce. The MICE industry in Beijing-Tianjin-Hebei Region (BTH) is unbalanced and led by the Yangtze River Delta and the Pearl River Delta [18,19]. Therefore, in the new new development stage, it is necessary to further understand the spatial structure characteristics and analyze the relevant factors affecting the spatial structure of the BTH MICE industry. Therefore, corresponding measures could be taken to jointly promote the coordinated development of the BTH MICE industry, and achieve higher quality development among the BTH MICE cities in the "14th Five Year Plan" period [20,21].

MICE research in China has been gradually developed with the declaration and holding of large-scale events, and contains several stages [22]. At the beginning, the research on exhibition mainly focused on the role of exhibition activities in driving domestic and foreign trade, which was not yet combined with urban development. Not until the successful declaration of Beijing Olympic Games in 2001 and Shanghai World Expo in 2002, the researches started to focus on the construction of exhibition venues and facilities, and the economic role of exhibition activities relating to urban construction began to receive attention. Scholars mainly summarize the development experience of foreign exhibition cities and explore the law of urban exhibition facilities construction, and some scholars begin to think about the economic pulling effect of exhibition on regional development [23–25]. With the successful holding of Beijing Olympic Games in 2008 and Shanghai World Expo in 2010, China’s MICE research entered into the developing stage, scholars continued paying attention to the economic effects of urban exhibition activities, and started to show interest in the spatial phenomenon of urban exhibition[26–32]. By the time when Winter Olympics was declared successfully in 2015, China’s MICE research entered the mature stage, and exhibition activities were closely integrated with urban development. The research methods have moved from single to multiple, and research methods such as gravity model and social network analysis have been enriched [33–35].

From the spatial perspective, MICE research can be categorized into national level and city level. At the national level, it is mainly related to development level, efficiency analysis using industrial data and spatio-temporal evolution research [36–40], including evaluating the spatial distribution of domestic exhibition industry from the new perspective of regional clusters [41], and constructing an evaluation model suitable for the pull coefficient of exhibition in China by combining the dynamic development trend of exhibition industry [42]. At the city level, the research mainly focuses on the industry development, competitiveness enhancement, and optimization of spatial structure layout [43–46], and less involves the analysis of attractiveness of MICE cities. The study area mainly focuses on first-tier cities such as Beijing, Shanghai, Guangzhou and Shenzhen and their surrounding areas [47–50].

With the regional driving effect of MICE activities being paid attention to, MICE research should continue to break through the city limitation and focus on regional linkage and its internal network analysis. In this paper, Beijing-Tianjin-Hebei Region is selected as the study area. By adopting relevant data in 2012 and 2018, this paper obtained the linkage between BTH MICE cities based on the theory of spatial structure and research methods including gravity model and social network analysis. Furthermore, ArcGIS 10.5 was used to analyze the spatial structure characteristics of the attractiveness of BTH MICE cities, and geographically weighted regression (GWR) was used to analyze the influence of the spatial structure of the attractiveness of MICE cities. It is expected that the research results could help promote the optimal allocation of regional MICE resources and high-quality development of MICE industry in BTH.

## Data and methods

### Study area and data source

**Fig 1:**
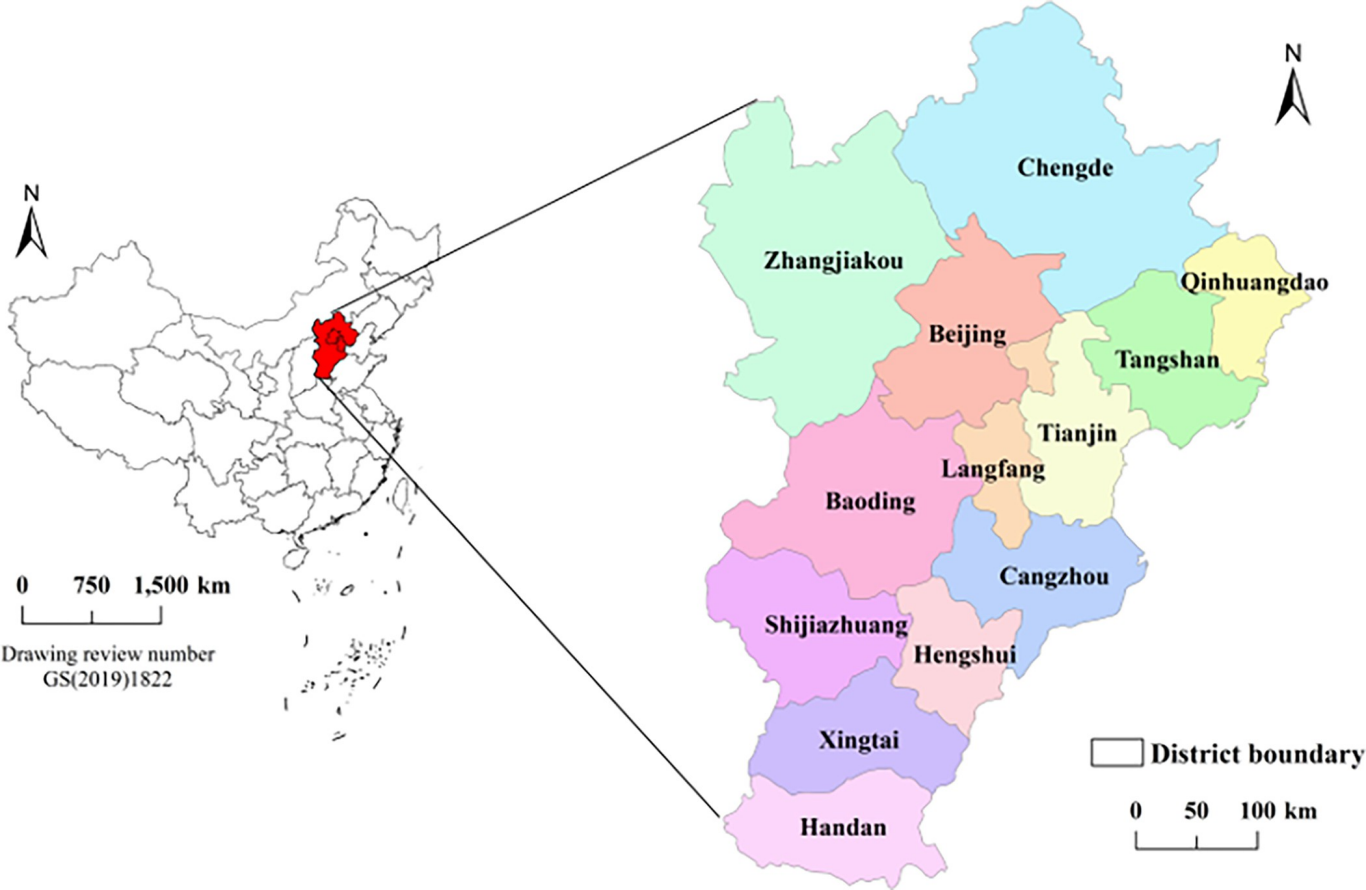
Map of the study area (Beijing, Tianjin and Hebei, China). Note: ArcGIS 10.5 is used for vector processing on the base map gained from the Ministry of Natural Resources, accessed November 17, 2021, at http://bzdt.ch.mnr.gov.cn/.

The study area is 13 prefecture level cities in Beijing, Tianjin and Hebei (see Fig 1), including Beijing, Tianjin, Langfang, Shijiazhuang, Hengshui, Tangshan, Xingtai, Handan, Zhangjiakou, Baoding, Cangzhou, Chengde and Qinhuangdao [51].

Referring to previous studies [30,33–34], this paper takes six variables, namely economic development level, population factors, tourism development level, urban environment, supporting facilities and Internet development level as the main influencing factors of the attractiveness of MICE cities in Beijing, Tianjin and Hebei. The basic calculation data used in the study are the MICE development index of Beijing, Tianjin and Hebei in 2012 and 2018, plus the shortest time used for railroad traffic in each city and 14 influencing factors (see Table 1), which come from the Statistics and Development Report of China Exhibition Industry generated by China Convention/Exhibition/Event Society (www.cces2006.org), the railroad traffic network (www.12306.cn), the Beijing Statistical Yearbook (http://tjj.beijing.gov.cn/), Tianjin Statistical Yearbook (http://stats.tj.gov.cn/) and Hebei Statistical Yearbook (http://tjj.hebei.gov.cn/).

**Table 1:** Influencing factors of MICE city attractiveness.

Explained variable	Explanatory variable	Indicator layer
**MICE city attractiveness** (A)	Economic development level (B1)	Added value of tertiary industry / 100 million yuan (C1)
		Foreign direct investment amount / USD 10000 (C2)
		Per capita GDP / yuan (C3)
	Population factors (B2)	Per capita disposable income / yuan (C4)
		Urbanization proportion of permanent residents (%) (C5)
		Number of permanent residents / 10000 (C6)
	Tourism development level (B3)	Total tourism reception revenue / 100 million yuan (C7)
		Total tourist reception / 10000 (C8)
	Urban environment (B4)	Fixed assets investment / 100 million yuan (C9)
		Urban green coverage rate (C10)
	Supporting facilities (B5)	Number of star rated hotels (C11)
		Fixed assets investment in leasing and business service industry / 100 million yuan (C12)
	Internet development level (B6)	Internet access users / 10000 households (C13)
		Fixed assets investment of communication industry / 100 million yuan (C14)

## Research methods

### Spatial structure analysis

#### Modified gravity model

Referring to the previous research on gravity model [52], a modified version could be adopted. The network space structure could be analyzed based on the attraction of MICE between two cities.


$$R_{\mathrm{ij}}=\frac{Z_{i}*Z_{j}}{D_{ij}}$$ (1)

Where: Zi and Zj are the MICE development indexes of the two cities respectively, and Dij is the shortest time (in hours) for railway traffic between two cities. R denotes the attractiveness of conventions among cities.

The attractiveness ranking of city i, can be obtained by summing the cities among the cities obtained from Eq (1), with the following equation.


$$\sum_{j=1}^{n-1}R_{ij}=R_{i1}+R_{i2}+R_{i3}+\ldots +R_{i(n-1)}$$ (2)

#### Network density analysis

It refers to the actual number of network relations divided by the theoretical maximum number of network relations, that is, it indicates the closeness of cities in the network. The network density value is within the range of 0–1. The closer the value is to 1, the higher the degree of connection between cities.


$$d(G)=\frac{2L}{n(n-1)}$$ (3)

Where: d(G) is the network density, n is the number of urban nodes, and L is the actual number of connected edges of the network.

#### Centrality analysis

Degree centrality, closeness centrality and betweenness centrality are selected to measure the status of nodes in the network. The degree centrality describes the communication ability of the nodes in the network. The higher the degree, the stronger the urban competitiveness. The closeness centrality reflects the ability of a node not to be controlled by surrounding nodes. The betweenness centrality belongs to the control ability index, which reflects the control power of a city over the spatial connection of other cities [53,54].


$$C_{D}(i)=\frac{D(i)}{n-1}$$ (4)

Where: *C*_*D*_(*i*) is the degree centrality of point i, *D*(*i*) is the number of effective connections between i city and other regions, and n is the number of urban nodes.


$$C_{C}(i)=\frac{n-1}{\sum_{j\neq 1}^{n}D_{\mathrm{ij}}}$$ (5)

Where: *C*_*C*_(*i*) is the closeness centrality of city i, *D*_*ij*_ is the shortest distance between city i and city j. The in-closeness centrality refers to the ease of other points reaching this point, the The out-closeness centrality refers to the ease of this point reaching other points, and n is the number of urban nodes.


$$C_{B}(i)=\frac{\frac{\sum_{j}^{n}\sum_{k}^{n}G_{jk}(i)}{G_{jk}}}{n^{2}-3n+2}(j\neq k\neq i,\;\mathrm{and}\;j<k)$$ (6)

Where: *C*_*B*_(*i*) is representing the betweenness centrality of city i; *G*_*jk*_(*i*) is representing the number of shortcuts passing through the point city i between the cities j and k; *G*_jk_ is represent the number of shortcuts from city j to city k, and n is the number of city nodes.

#### Cohesive subgroup analysis

Cohesive subgroup refers to the phenomenon of small group agglomeration formed through the internal relations of group members, which is conducive to displaying the characteristics of related cities and identifying the members with close and relatively stable relationships. The phenomenon of group agglomeration among the members of the subgroup indicates good interaction among subgroup members.

#### Core-edge structure analysis

By dividing the nodes in the network into two areas, namely the edge area and the core area, the closeness of the relationship between the nodes in the network could be identified, and the nodes in the core position and the edge position in the social network could be analyzed.

### Analysis on influencing factors

#### Normalized processing

Normalize the 14 influencing factors to make the data within the range of [0,1] and ensure the consistency of mathematical units. Through consulting relevant experts to assign values and quantify secondary indicators, GWR analysis can be carried out.


$$Y=\frac{X-M\mathrm{in}}{M\mathrm{ax}-M\mathrm{in}}$$ (7)

Where: Y represents the normalized value, X represents the initial value, Max represents the maximum value, and Min represents the minimum value.

The 14 selected influencing factors were normalized and assigned values to obtain the explanatory and explained variables for 2012 and 2018, and the GWR tool in ArcGIS 10.5 was used to construct the corresponding models, and the model bandwidth was calculated using the method AICc. The R^2^Adjusted was calculated to be 0.680 and 0.808 for 2012 and 2018. The R^2^Adjusted values range from 0 to 1. The larger the value, the better the fit of the model, namely the fit results are more desirable. Moran’s index was further calculated by entering the inverse-distance to obtain Moran’s I = 0.323567, Z = 2.289957, p = 0.022024 for 2012, indicating a 95% chance that the distribution is considered unlikely to be random and is clustered, therefore, the model is eligible overall. Moran’s I = 0.307264, Z = 2.23151, p = 0.025647 for 2018, indicating a acceptable overall effect [55]. This is the data fitting exercise prior to the geographically weighted regression, indicating that the overall data is valid.

#### Geographically weighted regression (GWR) analysis

The main advantage of GWR spatial linear regression model is that the spatial weight matrix is applied to the linear regression model, which can vividly show the spatial structure differentiation. It is one of the most potential spatial statistical models [56,57].


$$y_{i}=\beta_{0}(u_{i},v_{i})+\sum_{k=1}^{p}\beta_{k}(u_{i},v_{i})x_{ik}+\varepsilon_{i}$$ (8)

Where: y_i_ is the dependent variable, (*u*_*i*_, *v*_*i*_) is the geographic center coordinate of the i^th^ sample space unit, and *B*_k_(*u*_*i*_, *v*_*i*_) is the value of the connection function *β*_k_(*u*, *v*) in the i sample space unit.

## Results

### Analysis of MICE city relevance based on gravity model

The gravity model of the MICE city is formed by combining the attractiveness of the MICE cities and the traffic accessibility between cities. Through calculation, the attraction level of the MICE city is obtained (see Table 2), which reflects the changes of the urban attraction in the past seven years. According to Table 2, in 2012, only Beijing, Tianjin and Langfang had high attractiveness. In 2018, Beijing still accounted for a larger proportion and ranked first whereas other cities were more marginalized. In 2018, compared with 2012, the top seven cities in terms of attractiveness were relatively stable, while the bottom six cities did not fluctuate much and still accounted for a small proportion.

**Table 2:** Beijing-Tianjin-Hebei Region MICE city attractiveness ranking in 2012 and 2018.

	2012			2018		
Ranking	City	MICE Attractiveness	Proportion	City	MICE Attractiveness	Proportion
1	Beijing	34009.69	45.09%	Beijing	79407.95	47.44%
2	Langfang	19762.31	26.20%	Langfang	45208.34	27.01%
3	Tianjin	14610.26	19.37%	Tianjin	31646.23	18.90%
4	Tangshan	2098.07	2.78%	Tangshan	4036.28	2.41%
5	Shijiazhuang	1912.03	2.54%	Shijiazhuang	2752.34	1.64%
6	Cangzhou	1391.59	1.85%	Cangzhou	1640.45	0.98%
7	Xingtai	467.25	0.62%	Xingtai	1591.43	0.95%
8	Hengshui	281.59	0.37%	Qinhuangdao	360.24	0.22%
9	Baoding	276.57	0.37%	Baoding	245.92	0.15%
10	Qinhuangdao	219.91	0.29%	Zhangjiakou	206.34	0.12%
11	Zhangjiakou	195.32	0.26%	Chengde	116.32	0.07%
12	Handan	165.75	0.22%	Handan	99.91	0.06%
13	Chengde	31.25	0.04%	Hengshui	87.72	0.05%

### Social network space structure analysis

#### Network density analysis

**Fig 2:**
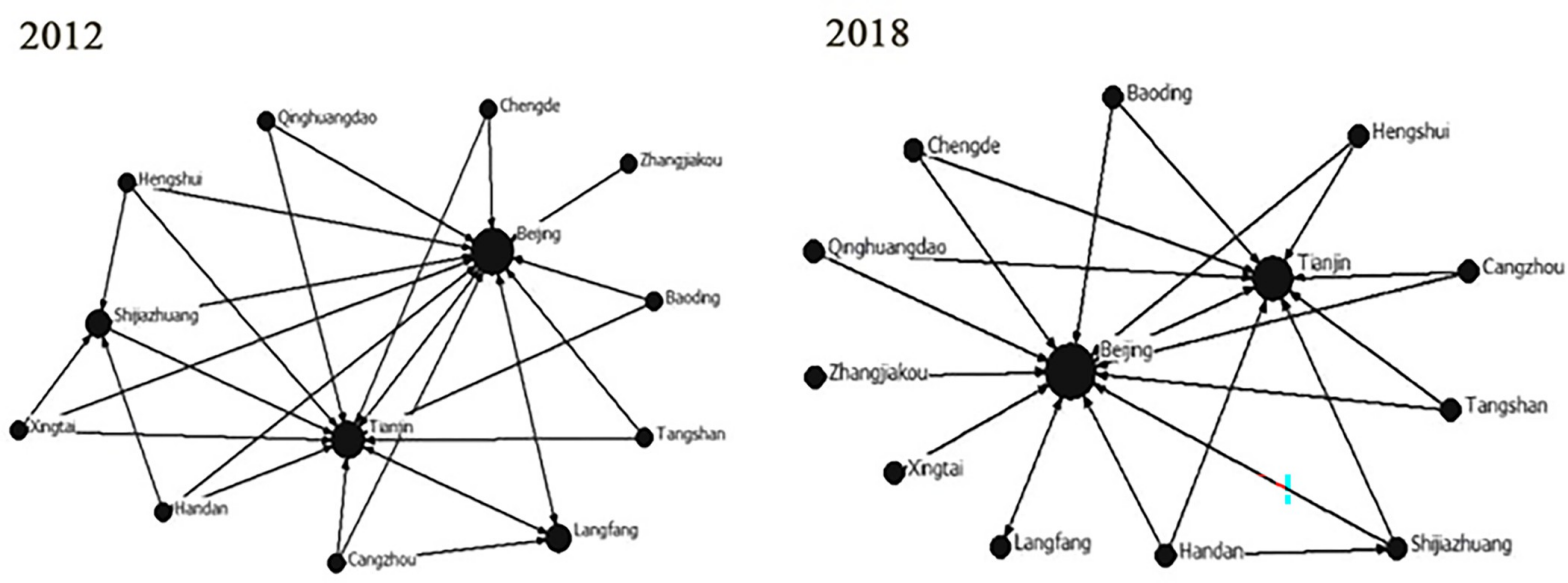
Network spatial structure of Beijing-Tianjin-Hebei MICE cities in 2012 and 2018.

Using Netdraw in UCINET, the network space structure map of the attractiveness of Beijing, Tianjin and Hebei MICE cities in 2012 and 2018 can be obtained (see Fig 2). In consideration of the comparability of the data, let Rij be the gravitational matrix between the cities. Then take $\bar{R}=\sum_{j=1}^{12}R_{ij}$ as the average value of each city of the gravitational matrix as the critical value. When $R_{\mathrm{ij}}>\bar{R}$, aij = 1, otherwise, aij = 0, and the adjacency matrix aij is obtained. Thus, the dichotomous matrix tables of 2012 and 2018 are obtained to represent the association network between MICE cities.

**Fig 3:**
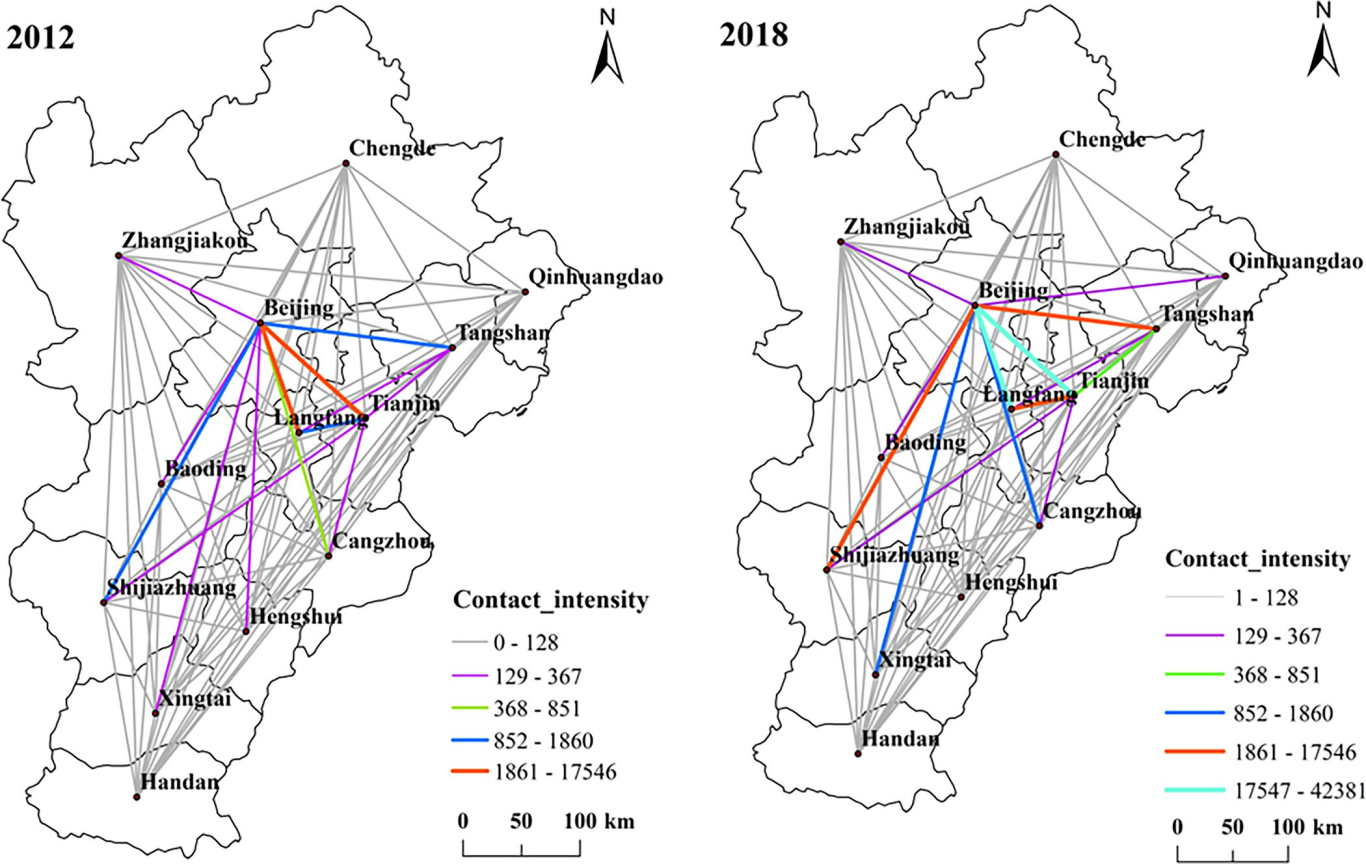
Contact intensity of Beijing-Tianjin-Hebei MICE cities in 2012 and 2018. Note: ArcGIS 10.5 is used for vector processing on the base map gained from the Ministry of Natural Resources, accessed November 17, 2021, at http://bzdt.ch.mnr.gov.cn/.

Using ArcGIS 10.5 to carry out spatial visual expression, and obtain the contact strength of Beijing-Tianjin-Hebei MICE cities in 2012 and 2018 (see Fig 3). From 2012 to 2018, the degree of MICE links has changed significantly with the passage of time. In 2012, the spatial structure of the degree of MICE cities in Beijing, Tianjin and Hebei spread around with Beijing as the center. Beijing, Tianjin and Langfang are interconnected, forming a closely linked core group, while Handan and Chengde have disadvantages in geographical location and transportation, which make the MICE industry underdeveloped and marginalized. In 2018, Beijing-Tianjin-Hebei MICE cities gradually gathered in Beijing, and the overall connection was significantly improved. The links between Beijing-Qinhuangdao, Beijing-Xingtai, Beijing-Shijiazhuang, Beijing-Cangzhou and Beijing-Tianjin were continuously strengthened, indicating that Beijing’s exhibition attraction continued to grow and its core position was stable. In general, due to the enhancement of Beijing’s own strength, it has strengthened the attraction of advantageous resources in the surrounding areas, enabling Beijing to play the largest radiation and driving role. However, the gap in the overall contact strength is gradually widening, indicating that the contact network of MICE cities is gradually becoming uneven.

The network density of MICE attraction of Beijing, Tianjin and Hebei cities is calculated by Formula (1), and the network density in 2012 and 2018 is 0.1859 and 0.1474 respectively, which is relatively low. Theoretically, in the network composed of 13 prefecture level cities, the maximum possible number of relationships is 156, while the actual number of relationships in the network in 2012 was only 29. Beijing and Tianjin have a large number of relationships, which are in the central position, followed by Shijiazhuang; In 2018, the number of actual relationships in the network decreased to 23, the number of relationships in Beijing increased, and the number of relationships in Tianjin and Shijiazhuang decreased.

#### Centrality analysis

Use Formula (4) to calculate the degree centrality of each city in 2012 and 2018 respectively. On the one hand, the penetration rates of Beijing and Tianjin in 2012 and 2018 were 12 and 11 respectively, far ahead in the degree of urban connection between Beijing, Tianjin and Hebei, which indicates that other cities have a large number of connections with these two cities, that is, these two cities have a strong attraction for MICE and can control and influence the flow of MICE resources in other cities. In 2018, the penetration of Langfang City and Shijiazhuang City decreased from 3 to 1, indicating that the attraction of MICE activities in the two cities has weakened. Tangshan City, Handan City, Xingtai City, Hengshui City, Zhangjiakou City, Cangzhou City, Baoding City, Qinhuangdao City and Chengde City have not yet got rid of the dilemma of zero penetration due to their lack of convention and exhibition resources or imperfect infrastructure. On the other hand, the average point out degree of each city in 2012 was 2.14, and the average point out degree of each city in 2018 was 1.67, and the deviation of point out degree in both years was 0.58. The low point out degree indicates that this city is less related to other cities, so it obtains less resources from other cities in the network. In general, the core regional position of Beijing is more prominent. The advantages of Beijing and Tianjin over other cities in Hebei are gradually expanding, and the spatial network shows a trend of unbalanced development.

Use Formula (5) to calculate the closeness centrality of cities in 2012 and 2018. The larger the value of the proximity to the center, the closer the relations between the city and other cities is. On the contrary, the more sparse the relation between the city and other cities is. From the longitudinal comparison of time, the point out degree of each city is rising, which indicates that the degree of each city affecting other cities is gradually increasing. The point in degree of Shijiazhuang City, Tangshan City and other nine cities is rising, which indicates that the degree of impact by other cities is increasing, while the point in degree of Beijing City, Tianjin City and Langfang City is decreasing, which indicates that it is not easy to be affected by other cities. From the horizontal comparison of various cities, in 2012, the penetration rate of Beijing, Tianjin and Langfang was far higher than that of other cities, indicating that these cities were more affected by other cities, while in 2018, the other nine cities, including Shijiazhuang and Tangshan, were more affected by other cities; In 2012, Handan, Xingtai and Hengshui had a larger point out degree than other cities, indicating that they had a greater impact on other cities. In 2018, Beijing, Tianjin and Langfang had a greater impact on other cities, and the point out degree of Beijing, Tianjin and Langfang was greater than the point in degree. It shows that these three cities affect other cities more than the rest cities.

Use Formula (6) to calculate the betweenness centrality of each city in 2012 and 2018. In 2012, the intermediate centrality of Beijing and Tianjin was 6 and 4 respectively, which indicates that one city has served as the shortest bridge between the other two cities more times, that is, the two cities have strong control ability in the regional network and grasp more convention and exhibition resources. The intermediate centrality of other cities is 0, which shows Beijing and Tianjin’s intermediate centrality is outstanding, and overall rather independent. In 2018, only Beijing’s intermediate centrality was valued at 14, which indicates that Beijing’s advantages are more prominent and it occupies a dominant position in the network centrality. Beijing plays a bridge and intermediary role in the connection of 13 cities. Therefore, Beijing, Tianjin and Hebei should strengthen their relations with each other to reduce Beijing’s core position to an agreeable degree. All the data mentioned above have been classified into Table 3 as follows.

**Table 3:** Centrality analysis in 2012 and 2018.

	2012					2018				
City	Degree centrality		Closeness centrality		Between -ness centrality	Degree centrality		Closeness centrality		Between -ness centrality
	In-degree	Out-degree	In-closeness	Out-closeness		In-degree	Out-degree	In-closeness	Out-closeness	
Beijing	12.00	2.00	100.00	9.09	6.00	12.00	2.00	12.00	132.00	14.00
Tianjin	11.00	2.00	92.31	9.09	4.00	9.00	1.00	15.00	133.00	0.00
Langfang	3.00	2.00	57.14	9.09	0.00	1.00	1.00	23.00	133.00	0.00
Shijiazhuang	3.00	2.00	10.00	9.92	0.00	1.00	2.00	144.00	121.00	0.00
Tangshan	0.00	2.00	7.69	9.92	0.00	0.00	2.00	156.00	121.00	0.00
Handan	0.00	3.00	7.69	11.01	0.00	0.00	3.00	156.00	109.00	0.00
Xingtai	0.00	3.00	7.69	11.01	0.00	0.00	1.00	156.00	122.00	0.00
Hengshui	0.00	3.00	7.69	11.01	0.00	0.00	2.00	156.00	121.00	0.00
Zhangjiakou	0.00	1.00	7.69	9.84	0.00	0.00	1.00	156.00	122.00	0.00
Cangzhou	0.00	3.00	7.69	10.00	0.00	0.00	2.00	156.00	121.00	0.00
Baoding	0.00	2.00	7.69	9.92	0.00	0.00	2.00	156.00	121.00	0.00
Qinhuangdao	0.00	2.00	7.69	9.92	0.00	0.00	2.00	156.00	121.00	0.00
Chengde	0.00	2.00	7.69	9.92	0.00	0.00	2.00	156.00	121.00	0.00

#### Cohesive subgroups analysis (CONGOR)

**Fig 4:**
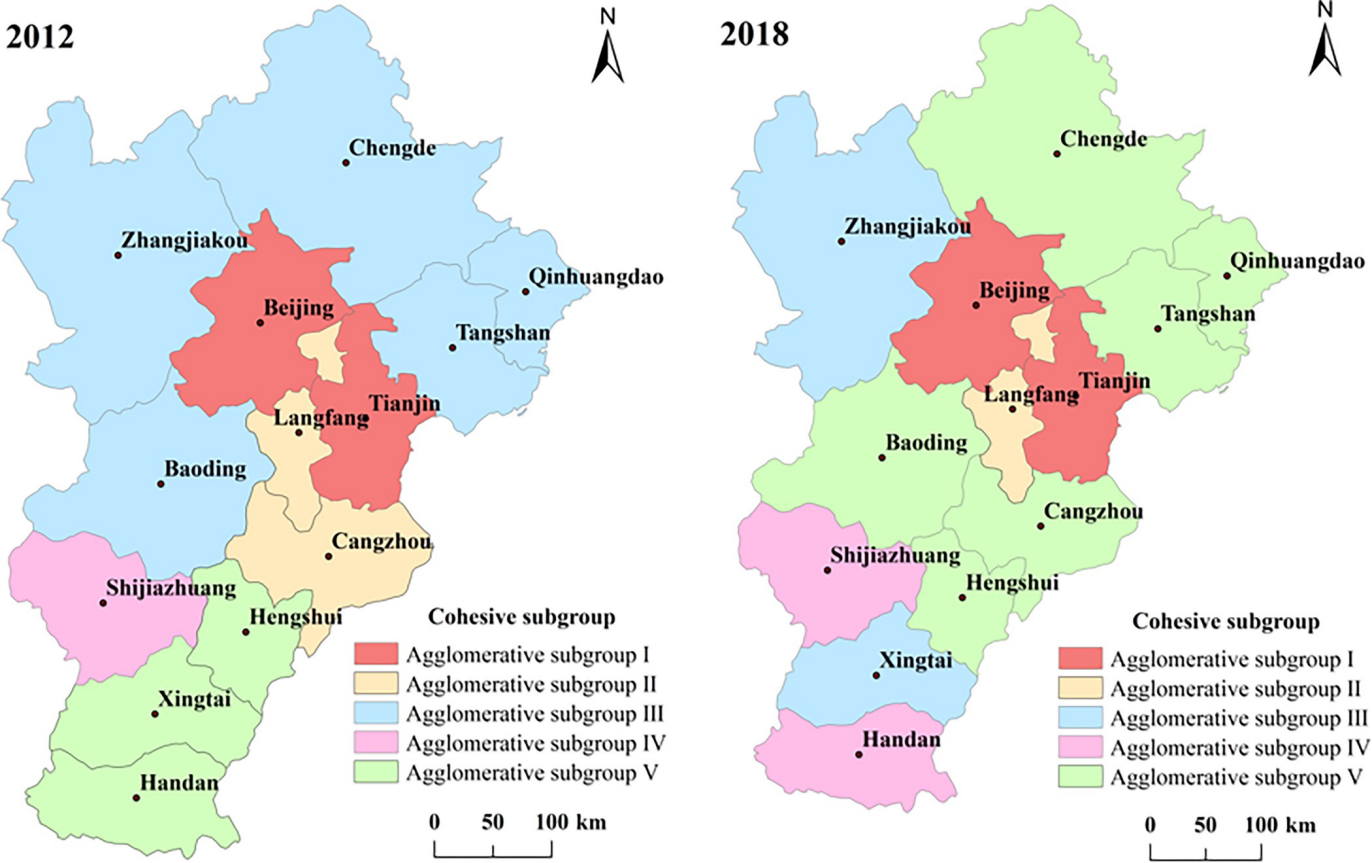
Analysis of cohesive subgroups in 2012 and 2018. Note: ArcGIS 10.5 is used for vector processing on the base map gained from the Ministry of Natural Resources, accessed November 17, 2021, at http://bzdt.ch.mnr.gov.cn/.

The cohesive subgroups of 2012 and 2018 were obtained by using UCINET software for cohesive subgroup analysis (see Fig 4). The I subgroup in 2012 is Beijing-Tianjin; The second subgroup is Langfang-Cangzhou; The third subgroup is Baoding-Chengde-Zhangjiakou-Tangshan-Qinhuangdao; The fourth subgroup is Shijiazhuang; The V subgroup is Hengshui-Xingtai-Handan, and there is no spatial fragmentation, which indicates that each subgroup has strong spatial self-organization ability and forms multiple independent subgroups. The I subgroup in 2018 is Beijing-Tianjin; The second subgroup is Langfang; The third subgroup is Xingtai-Zhangjiakou; The fourth subgroup is Handan-Shijiazhuang; The V subgroup is Hengshui-Baoding-Chengde-Cangzhou-Tangshan-Qinhuangdao. It can be seen that Beijing and Tianjin have always been clustered, and the subgroups formed by other cities have changed to some extent. In the past, the independent Shijiazhuang City and Handan City clustered together, while Langfang City became an independent subgroup and did not form a subgroup with other cities. This indicates that Langfang City has limited contact with other cities, and its internal spatial structure is gradually differentiate from other cities. There is a serious spatial fragmentation phenomenon in the whole. Subgroup V is divided by subgroups I, II and III, subgroup III is divided by subgroups V and IV, and subgroup IV is divided by subgroup III. Each subgroup is spatially separated from each other and acts as an intermediary channel for mutual exhibition.

#### Core-edge analysis

**Fig 5:**
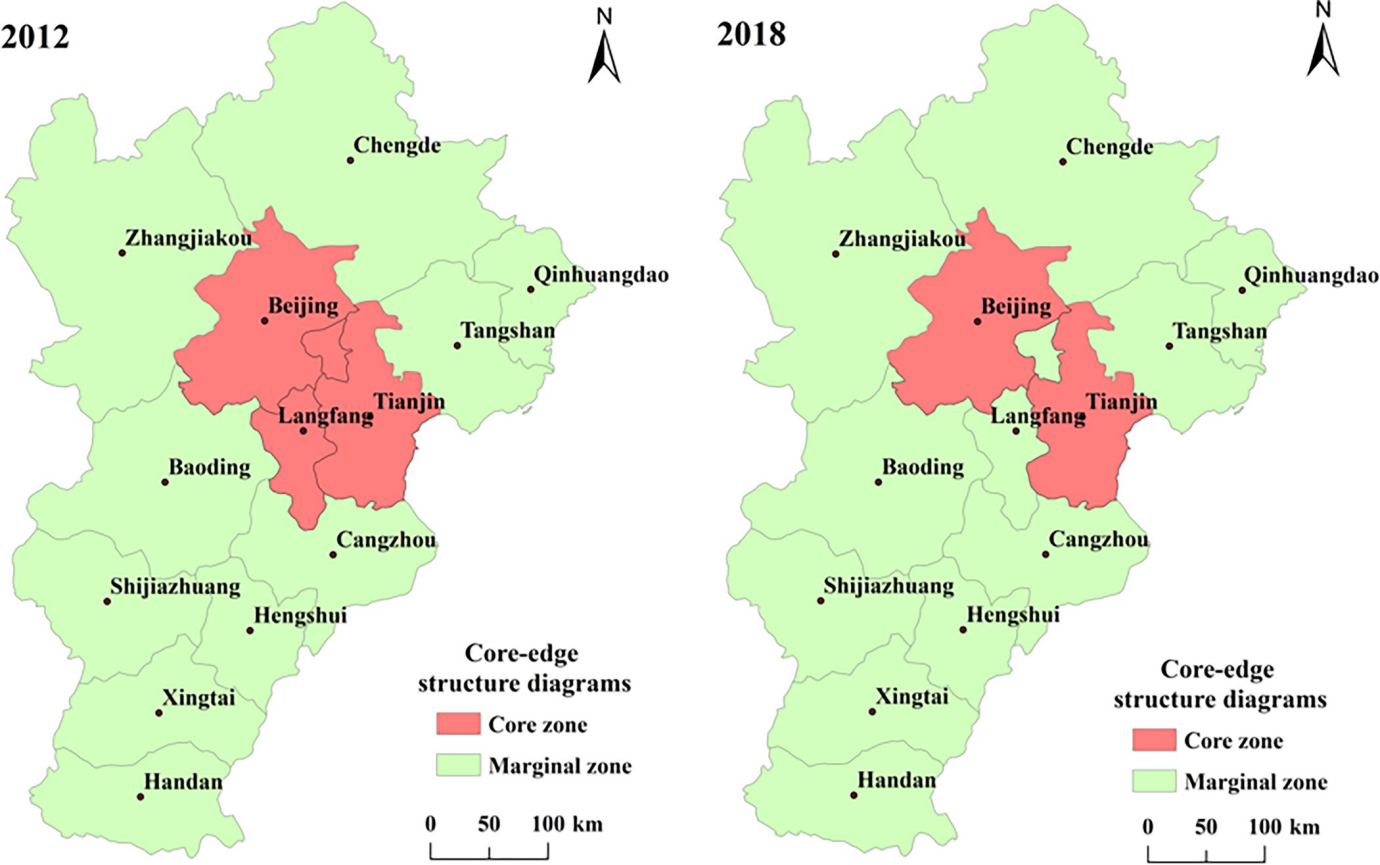
Core-edge structure diagrams in 2012 and 2018. Note: ArcGIS 10.5 is used for vector processing on the base map gained from the Ministry of Natural Resources, accessed November 17, 2021, at http://bzdt.ch.mnr.gov.cn/.

The core-edge structure in 2012 and 2018 was analyzed by UCINET software (see Fig 5). In 2012, the core areas were Beijing, Tianjin and Langfang, but by 2018, the core areas were only Beijing and Tianjin. The reason is the rapid economic development, population density, developed service industry and relatively perfect infrastructure of Beijing and Tianjin have led to a large number of exhibition resources gathering, and the attraction of Langfang’s MICE industry has decreased. Beijing and Tianjin have always been core areas, so they are classified as absolute core areas, while 10 cities such as Zhangjiakou, Chengde and Baoding belong to absolute marginal areas.

### Influencing factors of MICE city attractiveness in Beijing-Tianjin-Hebei

**Fig 6:**
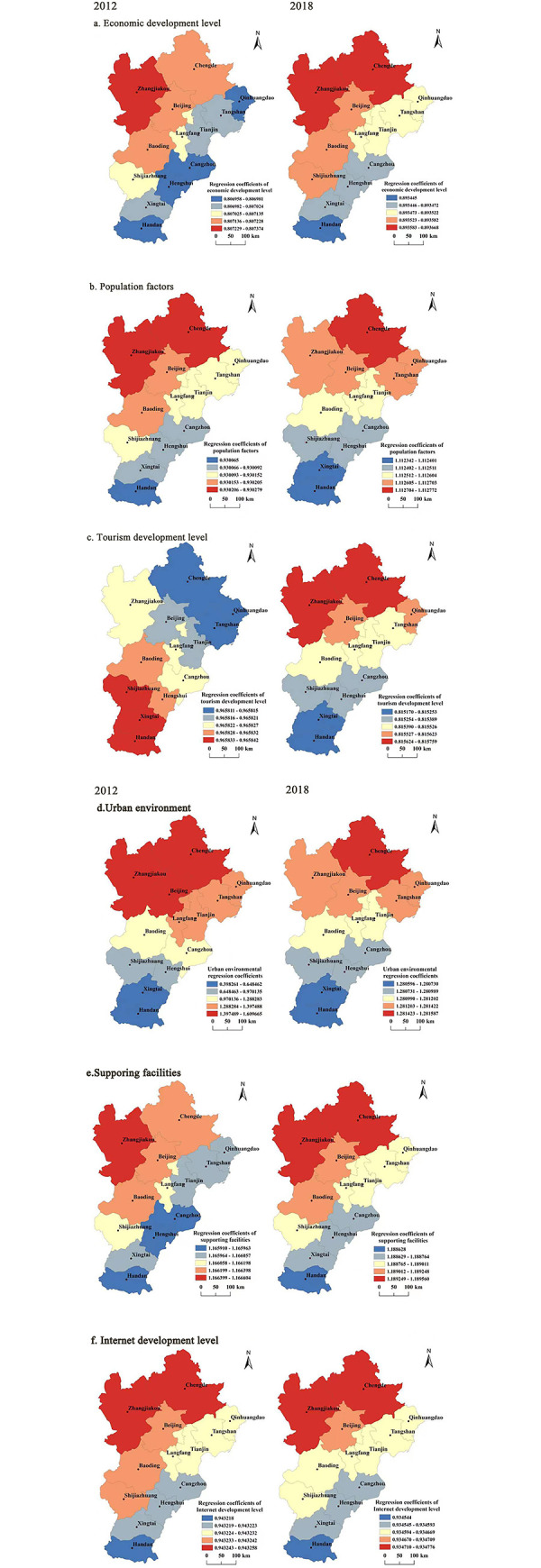
Spatial distribution of regression coefficients of spatio-temporal geographically weighted model in 2012 and 2018. Note: ArcGIS 10.5 is used for vector processing on the base map gained from the Ministry of Natural Resources, accessed November 17, 2021, at http://bzdt.ch.mnr.gov.cn/.

It is known from previous session that economic development level, population factors, tourism development level, urban environment, supporting facilities and Internet development level were selected as the main influencing factors to imply the attractiveness of MICE cities in Beijing, Tianjin and Hebei (see Table 1). In order to perform GWR model, the 14 selected influencing factors were normalized and assigned values to obtain the data of the explanatory and explained variables in 2012 and 2018, and the GWR tool in ArcGIS 10.5 was used to construct the corresponding models. The results are shown in Fig 6. Detailed explanations are as follows.

Fig 6A describes the spatial variation characteristics of the impact of economic development level on the attractiveness of MICE cities. The regression coefficients of economic development level in 2012 and 2018 are both positive, which are positively correlated with the attractiveness of MICE cities. From the spatial distribution of the regression coefficient, the regression coefficient in northwest of Beijing, Tianjin and Hebei was higher than that in the southeast from 2012 to 2018, and the regression coefficient in the south gradually decreased. This indicates the level of economic development has a great impact on the attraction of exhibitions in northern cities such as Beijing, Tianjin and Hebei, while the impact in the south is small.

Fig 6B shows the spatial variation characteristics of the impact of population factors on the attractiveness of MICE cities. In 2012 and 2018, the regression coefficient of population factors was positive, which was positively related to the attractiveness of MICE cities. From the spatial variation of the regression coefficient, In 2012, the spatial distribution of the regression coefficient gradually increased from south to north. Among them, the regression coefficient of Chengde and Zhangjiakou was larger, indicating that the impact of population factors on the attractiveness of MICE cities was greater in Chengde and Zhangjiakou. From the change of the mean value of the regression coefficient of the GWR Model, the influence of the population factor on the attractiveness of the MICE city is increasing.

Fig 6C draws the spatial variation characteristics of the impact of tourism development level on the attractiveness of MICE cities. In 2012 and 2018, The regression coefficients of tourism development level are all positive, which are positively related to the attractiveness of MICE cities. From the perspective of the spatial change of the regression coefficient, the spatial distribution of the regression coefficient in 2012 showed a decreasing trend from south to north. The impact of tourism development level on the attractiveness of MICE cities was the largest in Handan and Shijiazhuang; In 2018, the overall spatial distribution of the regression coefficient showed a trend of gradually increasing from south to north. The impact of tourism development level on the attractiveness of MICE cities was the largest in Qinhuangdao and Zhangjiakou. From the change of the mean value of the regression coefficient of GWR Model, the impact of tourism development level on the attractiveness of MICE cities is decreasing.

Fig 6D is the spatial variation characteristics of the impact of urban environment on the attractiveness of MICE cities. In 2012 and 2018, the urban environment regression coefficient was positive, and the urban environment was positively related to the attractiveness of the MICE city. From the perspective of spatial distribution, the overall regression coefficient in the North was high in 2012 and 2018, and the coefficient in the South gradually decreased, indicating that the urban environment has a great impact on the attraction of exhibitions in Zhangjiakou and Chengde. From the change of the mean value of the regression coefficient of the GWR Model, from 2012 to 2018, the impact of the urban environment on the attractiveness of Beijing-Tianjin-Hebei MICE cities were gradually increased.

Fig 6E shows the spatial variation characteristics of the impact of supporting facilities on the attractiveness of MICE cities. In 2012 and 2018, the regression coefficient of supporting facilities was positive, which was positively related to the attractiveness of MICE cities. From the perspective of the spatial change of the regression coefficient, the overall spatial distribution of the regression coefficient from 2012 to 2018 gradually decreases from the northwest to the southeast. The impact of supporting facilities on the attractiveness of the MICE city is the largest in Zhangjiakou. From the change of the mean value of the regression coefficient of the GWR Model, the influence of supporting facilities on the attractiveness of the MICE city is increasing.

Fig 6F describes the spatial variation characteristics of the impact of Internet development level on the attractiveness of MICE cities. In 2012 and 2018, the regression coefficient of Internet development level was positive, which was positively related to the attractiveness of MICE cities. From the perspective of the spatial change of the regression coefficient, the overall spatial distribution of the regression coefficient from 2012 to 2018 gradually decreases from the northwest to the southeast. The impact of the Internet development level on the attractiveness of MICE cities is greatest in Zhangjiakou and Qinhuangdao.

## Discussion

First, in 2012 and 2018, the network spatial structure of the Beijing-Tianjin-Hebei MICE cities spread around with Beijing as the center. Beijing, Tianjin and Langfang formed a closely linked core group, and Handan and Chengde were gradually marginalized. Due to the enhancement of Beijing’s core role, Beijing has strengthened its attraction to the advantageous resources in the surrounding areas, but the overall MICE city network has gradually become unbalanced.

Second, from the perspective of degree centrality, from 2012 to 2018, Beijing and Tianjin had strong attraction for MICE, which could control and influence the flow of resources in other cities, while Langfang and Shijiazhuang had weak attraction for MICE. In the network, each city obtains less resources from other cities, which shows that Beijing’s core position in the region is more prominent. The advantages of Beijing and Tianjin over Hebei cities are gradually expanding, and the spatial network shows a trend of unbalanced development. From the perspective of closeness centrality, the impact of 13 cities in Beijing, Tianjin and Hebei on other cities is gradually increasing, while the impact of other cities on the other 9 cities such as Shijiazhuang and Tangshan is increasing, while the impact of other cities on Beijing, Tianjin and Langfang is decreasing. From the perspective of betweenness centrality, in 2012, the middle centrality of Beijing and Tianjin was prominent and the overall situation was independent; In 2018, the advantages of Beijing will become more prominent and occupy a dominant position in the network centrality. Beijing plays a bridge and intermediary role in the connection of 13 cities. Therefore, the Beijing-Tianjin-Hebei urban MICE should strengthen the relationship between each other to appropriately reduce Beijing’s core position.

Third, from 2012 to 2018, the overall space has formed from a single independent subgroup to a subgroup that is spatially separated from each other and also serves as an intermediary channel for each other. Beijing and Tianjin are absolute core areas, while Zhangjiakou, Chengde and Baoding are absolute marginal areas. Due to the rapid economic development, dense population and developed service industry in Beijing and Tianjin, a large number of exhibition resources are concentrated on Beijing and Tianjin, and the attraction of MICE industry in Langfang is reduced, that is, the number of core areas is reduced from 3 to 2.

Fourth, from 2012 to 2018, the impact of supporting facilities, urban environment and population factors on the attractiveness of Beijing-Tianjin-Hebei MICE cities were gradually increased, and the impact of tourism development level on the attractiveness of Beijing-Tianjin-Hebei MICE cities were gradually decreased, and the impact of economic development level and Internet development level on the attractiveness of Beijing-Tianjin-Hebei MICE cities remained unchanged. The level of economic development, population factors, urban environment, supporting facilities and Internet development have a great impact on the attraction of MICE in the north, especially Chengde and Zhangjiakou, while the impact in the south is small. The level of tourism development has gradually changed from having a great impact on the attraction of MICE in Handan and Shijiazhuang to having a great impact on the attraction of MICE in Qinhuangdao and Zhangjiakou.

## Conclusions

Given the research results, the core role of Beijing, Tianjin and Langfang should be brought into play to drive the development of the neighboring MICE cities, establish a link between Beijing, Tianjin and Hebei MICE cities. The BTH cooperation strengthens the construction of transportation infrastructure between Beijing, Tianjin and Hebei, reduces the cost of flow of factor resources in the network of MICE cities, optimizes the allocation of resources in the city network of BTH MICE industry, enhances the economic external effect of MICE cities and provides the allocation efficiency of exhibition resources.

While Beijing, Tianjin and Langfang focus on promoting, the exhibition resources of Beijing-Tianjin-Hebei Region are integrated through the organization and coordination of government departments to realize the sharing and effective use of resources. It fully uses Internet technology and big data analysis technology to strengthen the communication and sharing of Beijing-Tianjin-Hebei Region’s MICE development in terms of venues, projects and talents, and strengthen the cooperation among related enterprises. Ultimately, it realizes balanced development within the region, avoids homogeneous competition and advocates complementary development.

In order to realize the scale economy and regionalization economy of MICE industry in these core cities, Beijing, Tianjin and Langfang need to cultivate a number of world-class exhibitions. At the same time, developing special exhibition resources of other cities cannot be overlooked. By promoting the regional integration of MICE market and encourage joint exhibition forms, the urban network pattern of Beijing-Tianjin-Hebei Region’s MICE industry becomes an organic whole of synergistic linkage.

There are some shortcomings in the article. Due to the in-comprehensiveness of statistical information and data availability restricting the access to some related industry indicators, data from more segmented industries can be used as variables in the future to make the research results more realistic. Less consideration is given to the impact of the new crown epidemic, and the subsequent study should consider the impact of the unexpected events on the MICE industry and related industries to make the model more suitable for reality. The MICE industry is essentially the gathering and dispersal of people and logistics, so the supporting facilities and transportation infrastructure of the city will have an impact on the development of urban MICE, and the introduction of the above two variables should be considered in the future.

## References

[1] Zhou L, Zhou CH, Chen L, Wang B. Spatio-temporal Evolution and Influencing Factors of Urban Green Development Efficiency in China. Journal of Geographical Sciences, 2020, 30(5): 724–742. doi: 10.1007/s11442-020-1752-5

[2] Gong PM, Gong PH, Zhang HF. The Evolution and Reform Orientation of Beijing Tianjin Hebei Coordinated Development Strategy. Regional Economic Review, 2020, (6): 63–70. doi: 10.14017/j.cnki.2095-5766.2020.0110

[3] Zheng MR, Zheng XQ, Li TL, Zhang LL, Lyu YQ. Big-data driven functional interaction patterns and governance strategy for Beijing-Tianjin-Hebei region. Acta Geographic Sinica, 2022, 77(6): 1374–1390. doi: 10.11821/dlxb202206006

[4] Hu HW, Wang JR. A Research on Government-driven Governance in the Collaboration of Elderly Care Services among the Beijing-Tianjin-Hebei Region-From the Perspective of Organizational Multiple Institutional Logic. Journal of Beijing Union University(Humanities and Social Sciences), 2022, 20(1): 60–69. doi: 10.16255/j.cnki.11-5117c.2022.0009

[5] Chen H, Luo LF. Impact of Regional Coordinated Development Policy on Factor Flow and Configuration: Taking Beijing-Tianjin-Hebei Region as an Example. Reform: 1–19 [cited 2023 February 2]. Available from:http://kns.cnki.net/kcms/detail/50.1012.F.20221019.1630.002.html.

[6] Li HF. Investigating the Topics Discovery and Correlation Analysis of Newspaper Reports on the Integrated Development of Beijing-Tianjin-Hebei Region. Science Technology and Engineering, 2021, 21(28): 12185–12193. doi: 10.3969/j.issn.1671-1815.2021.28.035

[7] Zhang MY, Bian XY. Evaluation of the Critical Effect of Coordinated Development Policy in the Beijing-Tianjin-Hebei Region Based on the Regression Discontinuity. Regional Economic Review, 2022, (4): 45–52. doi: 10.14017/j.cnki.2095-5766.2022.0072

[8] Wang SH, Yang ZW, Zhang W, Liu Y. Measurement and obstacle factor analysis of industrial green coordinated development in Beijing-Tianjin-Hebei. Journal Of Statistics And Information, 2022, 37(1): 34–44. doi: 10.3969/j.issn.1007-3116.2022.01.004

[9] Li HF, Xu JM, Li JK. Literature Characteristics and Hotspots of Coordinated Development of Beijing-Tianjin-Hebei-Based on Bibliometric and Co-word Analysis. Journal of Hebei University(Philosophy and Social Science), 2019, 44(1): 97–107. doi: 10.3969/j.issn.1005-6378.2019.01.014

[10] Yang ZY. Public perception and effect evaluation of the coordinated development of the Beijing-Tianjin-Hebei Region: Based on field observations in four districts and counties. Hubei Social Sciences, 2022, (1): 55–62. doi: 10.13660/j.cnki.42-1112/c.015784

[11] Zhou L, Hu FN, Wang B, Wei CZ, Sun DQ, Wang SH. Relationship between urban landscape structure and land surface temperature: spatial hierarchy and interaction effects. Sustainable Cities and Society, 2022. doi: 10.1016/J.SCS.2022.103795

[12] Manuela G, Claudia G, Samuel B, Gelsomina F, Daniela L. A botanic garden as a tool to combine public perception of nature and life-science investigations on native/exotic plants interactions with local pollinators. Plos One, 2020, 15(2). doi: 10.1371/journal.pone.0228965 32078664 PMC7032708

[13] Anas A, Arnott R, Small KA. Urban Spatial Structure. Journal of Economic Literature, 1998, 36(3).

[14] Gu RD, Li CF, Li DD, Yang YY, Gu S. The Impact of Rationalization and Upgrading of Industrial Structure on Carbon Emissions in the Beijing-Tianjin-Hebei Urban Agglomeration. International Journal of Environmental Research and Public Health, 2022, 19(13). doi: 10.3390/ijerph19137997 35805656 PMC9265910

[15] Yang ZS, Yang H, Wang H. Evaluating urban sustainability under different development pathways: A case study of the Beijing-Tianjin-Hebei region. Sustainable Cities and Society, 2020, 61. doi: 10.1016/j.scs.2020.102226

[16] Volgmann K, Rusche K. The Geography of Borrowing Size: Exploring Spatial Distributions for German Urban Regions. Tijdschrift Voor Economische En Sociale Geografie, 2020, 111(1). doi: 10.1111/tesg.12362

[17] Li CX, Gao X, He BJ, Wu JY, Wu KN. Coupling Coordination Relationships between Urban-industrial Land Use Efficiency and Accessibility of Highway Networks: Evidence from Beijing-Tianjin-Hebei Urban Agglomeration, China. Sustainability, 2019, 11(5). doi: 10.3390/su11051446

[18] Zhou L, Tian L, Cao YD, Yang LC. Industrial land supply at different technological intensities and its contribution to economic growth in China: A case study of the Beijing-Tianjin-Hebei region. Land Use Policy, 2020. doi: 10.1016/j.landusepol.2020.105087

[19] Li WH, Song HF, Dong F, Li FF. The high-quality development in Beijing-Tianjin-Hebei regions: Based on the perspective of comparison. Procedia Computer Science, 2022, 199. doi: 10.1016/J.PROCS.2022.01.158

[20] Luo YM, Zhou DY, Tian YY, Jiang GH. Spatial and temporal characteristics of different types of pollution intensive industries in the Beijing-Tianjin-Hebei region in China by using land use data. Journal of Cleaner Production, 2021, 329. doi: 10.1016/J.JCLEPRO.2021.129601

[21] Wang KP, Wang WQ, Zha NY, Feng Y, Qiu CL, Zhang YL, et al. Spatially Heterogeneity Response of Critical Ecosystem Service Capacity to Address Regional Development Risks to Rapid Urbanization: The Case of Beijing-Tianjin-Hebei Urban Agglomeration in China. Sustainability, 2022, 14(12). doi: 10.3390/SU14127198

[22] Li T, Ye QQ, Chen QX. Research on the transformation from international exhibition to cloud exhibition in the post COVID-19 era: A case study of China International Fair for Investment & Trade. Plos One, 2022, 17(4). doi: 10.1371/JOURNAL.PONE.0267455 35482809 PMC9049316

[23] Liu DY. Exploring the Productivization of Shanghai’s Tourism Resources. Tourism Tribune, 2000,15(04):38–42. doi: 10.3969/j.issn.1002-5006.2000.04.008

[24] Ma Y. On the Development of Chinese Mice Economy. Economic Geography, 2002, (3): 293–296. doi: 10.3969/j.issn.1000-8462.2002.03.010

[25] Zhu HS. Study and Inspiration on the Spatial Distribution of Overseas MICE Industry-Taking Germany and Hong Kong as Examples. Human Geography, 2004, (5): 93–96. doi: 10.13959/j.issn.1003-2398.2004.05.021

[26] Peng XQ, Wang JE. An Analysis of the Space-time and Industrial Features of Professional Exhibitions in China. Tourism Tribune, 2008, (9): 80–84. doi: 10.3969/j.issn.1002-5006.2008.09.020

[27] Shen DY, Chen ZY. A review of the theory of exhibition economy in China. Commercial Research, 2009, (9): 111–115. doi: 10.3969/j.issn.1001-148X.2009.09.033

[28] Guo JR. Thinking about Chinese exhibition economy development opportunities. Shanghai Management Science, 2013, 35(4): 82–84. doi: 10.3969/j.issn.1005-9679.2013.04.018

[29] Cong L, Wu BH, Kou X. Research on Spatial Distribution of Convention Industry in Beijing. Economic Geography, 2013, 33(5): 77–83. doi: 10.15957/j.cnki.jjdl.2013.05.013

[30] Xiao YN, Zhang XH, Li X. Research on Regional Cooperation Mechanism of Conference and Exhibition Industry in Pearl River Delta Urban Agglomerations: Based on city attraction model. Reform of Economic System, 2012, (1): 57–60. Available from:https://kns.cnki.net/kcms/detail/detail.aspx?dbname=CJFD2012&filename=JJTG201201015&dbcode=CJFD.

[31] Fang ZQ, Wang ZJ, Liu L. Analysis on the Change of the Spatial Pattern of Exhibition Enterprises in the Pearl River Delta. China Population, Resources and Environment, 2013, 23(7): 149–154. doi: 10,3969/j.issn.1002-2104.2013.07.023

[32] Ren GY. The Agglomeration Characteristics and Influencing Factors of Exhibition Venues in Yangtze River Delta. Economic Geography, 2014, 34(9): 86–92. doi: 10.15957/j.cnki.jjdl.2014.09.043

[33] Cai WM, Zeng M, Li DJ. Spatial-Temporal Characteristics and Influencing Factors of Tourism Microblogs to China’s MICE City Based on Sina Microblog Data. Economic Geography, 2020, 40(8): 185–193. doi: 10.15957/j.cnki.jjdl.2020.08.023

[34] Zhang HZ, Lu L. Study on Coordinated Development of MICE Industry and Urban Cities: A Case of Beijing-Tianjin-Hebei Metropolitan Area and Yangtze River Delta. Areal Research and Development, 2017, 36(3): 46–54. Available from:https://kns.cnki.net/kcms/detail/detail.aspx?dbname=CJFD2017&filename=DYYY201703009&dbcode=CJFD.

[35] Lin L, Li J. The spatial correlation effect and policy implications of China’s exhibition development—based on social network analysis method. Hebei Academic Journal, 2020, 40(1): 162–167. Available from:https://kns.cnki.net/kcms/detail/detail.aspx?dbname=DKFX2020&filename=HEAR202001021&dbcode=DKFX.

[36] Liu DJ, Chen JZ, Jai YY. Spatial Pattern and Influential Factors of Exhibition Passenger Flow in China. Economic Geography, 2019, 39(12): 103–109. doi: 10.15957/j.cnki.jjdl.2019.12.012

[37] Liu Z, Lou JJ. Evaluation on the development of urban exhibition industry in China. Urban Problems, 2018, (6): 51–60. doi: 10.13239/j.bjsshkxy.cswt.180607

[38] Yan HL, He B, Xu F, Bai J. The Research on Exhibition Industry Efficiency of Chinese Capital City based on Data Envelopment Analysis. Tourism Forum, 2018, 11(3): 17–24. doi: 10.15962/j.cnki.tourismforum.201803025

[39] Wang QJ, Huang M, Yao J. Spatial Evolution Characteristics and Coupling Relationship of Exhibition Industries in China from the Perspective of Performance. Geography and Geo-Information Science, 2020, 36(1): 136–142. doi: 10.3969/j.issn.1672-0504.2020.01.020

[40] Yang X, Jin LM. The Spatio-Temporal Characteristics of Exhibition Industry in China. Economic Geography, 2014, 34(8): 96–102. doi: 10.15957/j.cnki.jjdl.2014.08.043

[41] Li WX, Yu D. An Empirical Research on the Spatial Distribution of Exhibition Industry in China Based on Geography. Journal of Capital University of Economics and Business, 2011, 13(5): 40–46. doi: 10.3969/j.issn.1008-2700.2011.05.007

[42] Niu PQ, Zhang M, Li BQ. Research on the evaluation model of Pulling coefficient of Chinese exhibition industry. Modern Management Science, 2015, No.266(05):100–102. doi: 10.3969/j.issn.1007-368X.2015.05.033

[43] Wang PL, Cai ML, Peng PG. Research on the development path of exhibition Industry in small and medium-sized cities—taking Chenzhou as an example. Social Sciences in Hunan, 2018, (4): 152–158. Available from:https://kns.cnki.net/kcms/detail/detail.aspx?dbname=CJFD2018&filename=FLSH201804022&dbcode=CJFD.

[44] Yu GX, Dai GQ. The Negative Effects of the Iconic Government-operating Expositionsto the Development of City Exhibition Industry-A Case Study of the Canton Fair. Contemporary Economic Management, 2017, 39(1): 66–72. doi: 10.13253/j.cnki.ddjjgl.2017.01.011

[45] Chai SS, Bao H, Chang HL. A research on the spatial distribution and function optimization of urban MICE industry based on the location and spatial structure theory. Journal of Qingdao University of Science and Technology (Social Sciences), 2010, 26(3): 21–26. doi: 10.3969/j.issn.1671-8372.2010.03.005

[46] Ju SL, Lu L, Yang XZ, Zhu TX. Research on the Spatial Structure of China’s Exhibition Economy Based on Urban System. Urabn Planning Forum, 2006, (1): 93–97. Available from:https://kns.cnki.net/kcms/detail/detail.aspx?dbname=cjfd2006&filename=CXGH200601018&dbcode=cjfd.

[47] Fang ZQ. The agglomeration characteristics and influencing factors of exhibition enterprises in Guangzhou. Acta Geographica Sinica, 2014, 68(04):464–476. Available from:https://kns.cnki.net/kcms/detail/detail.aspx?FileName=DLXB201304007&DbName=CJFQ2013.

[48] Wang CC, Zhou Y. The key elements for Beijing to build an international convention center city. Urban Problems, 2014, (11): 39–43. doi: 10.13239/j.bjsshkxy.cswt.141107

[49] Li CX, Wu KN. An input–output analysis of transportation equipment manufacturing industrial transfer: Evidence from Beijing‐Tianjin‐Hebei region, China. Growth and Change, 2021, 53(1). doi: 10.1111/GROW.12571

[50] Li CX, Wu KN, Gao XY. Manufacturing industry agglomeration and spatial clustering: Evidence from Hebei Province, China. Environment, Development and Sustainability: a Multidisciplinary Approach to the Theory and Practice of Sustainable Development, 2020, 22(8). doi: 10.1007/s10668-019-00328-1

[51] Zhang PY, Yang D, Qin MZ, Jing WL. Spatial heterogeneity analysis and driving forces exploring of built-up land development intensity in Chinese prefecture-level cities and implications for future Urban Land intensive use. Land Use Policy, 2020, 99. doi: 10.1016/j.landusepol.2020.104958

[52] Li J, Chen S, Wan GH, Fu CM. Study on the Spatial Correlation and Explanation of Regional Economic Growth in China-Based on Analytic Network Process. Economic Research Journal, 2014, 49(11): 4–16. Available from:https://kns.cnki.net/kcms/detail/detail.aspx?dbname=CJFD2014&filename=JJYJ201411002&dbcode=CJFD.

[53] Rong TQ, Zhang PY, Zhu HR, Jiang L, Li YY, Liu ZY. Spatial correlation evolution and prediction scenario of land use carbon emissions in China. Ecological Informatics, 2022, 71. doi: 10.1016/J.ECOINF.2022.101802

[54] Feng XH, Xiu CL, Zhong YX, Li ZR. The network hierarchy of cities in the Yangtze River Economic Belt—Based on the perspective of railway and highway passenger transport. Urban Problems, 2017, (7): 18–26. doi: 10.13239/j.bjsshkxy.cswt.170703

[55] Zhang J, Zhang LF, Pu LJ, Guan CM. Research on Spatio-temporal Variation of Urban Residential Land Price based on GWR Model: A Case Study of Jiangsu Province. Scientia Geographic Sinica, 2012, 32(7): 828–834. doi: 10.13249/j.cnki.sgs.2012.07.008

[56] Yang D, Zhang PY, Jiang L, Zhang Y, Liu ZY, Rong TQ. Spatial change and scale dependence of built-up land expansion and landscape pattern evolution—Case study of affected area of the lower Yellow River. Ecological Indicators, 2022, 141. doi: 10.1016/J.ECOLIND.2022.109123

[57] Wang RY, Huang BS, Pan ZL, Liu Y. Spatial pattern evolvement of the distribution of foodborne illness in China and its influence mechanism. World Regional Studies, 2020, 29(1): 168–180. doi: 10.3969/j.issn.1004-9479.2020.01.2018432

